# Transcriptome profiling of A549 non-small cell lung cancer cells in response to *Trichinella spiralis* muscle larvae excretory/secretory products

**DOI:** 10.3389/fvets.2023.1208538

**Published:** 2023-07-31

**Authors:** Haoxuan Wang, Yingying Zhu, Meichen Li, Jingdan Pan, Dan Li, Wen-Ping Guo, Guangcheng Xie, Luanying Du

**Affiliations:** ^1^Department of Pathogenic Biology, Chengde Medical University, Chengde, Hebei, China; ^2^Department of Clinical Laboratory, First Hospital of Qinhuangdao, Qinhuangdao, Hebei, China; ^3^Department of Laboratory, North China University of Science and Technology Affiliated Hospital, Tangshan, Hebei, China

**Keywords:** *Trichinella spiralis*, excretory/secretory products, transcriptome, glucose metabolism, non-small cell lung cancer A549 cells

## Abstract

*Trichinella spiralis* (*T. spiralis*) muscle-larva excretory/secretory products (ML-ESPs) is a complex array of proteins with antitumor activity. We previously demonstrated that ML-ESPs inhibit the proliferation of A549 non-small cell lung cancer (NSCLC) cell line. However, the mechanism of ML-ESPs against A549 cells, especially on the transcriptional level, remains unknow. In this study, we systematically investigated a global profile bioinformatics analysis of transcriptional response of A549 cells treated with ML-ESPs. And then, we further explored the transcriptional regulation of genes related to glucose metabolism in A549 cells by ML-ESPs. The results showed that ML-ESPs altered the expression of 2,860 genes (1,634 upregulated and 1,226 downregulated). GO and KEGG analysis demonstrated that differentially expressed genes (DEGs) were mainly associated with pathway in cancer and metabolic process. The downregulated genes interaction network of metabolic process is mainly associated with glucose metabolism. Furthermore, the expression of phosphofructokinase muscle (PFKM), phosphofructokinase liver (PFKL), enolase 2 (ENO2), lactate dehydrogenase B (LDHB), 6-phosphogluconolactonase (6PGL), ribulose-phosphate-3-epimerase (PRE), transketolase (TKT), transaldolase 1 (TALDO1), which genes mainly regulate glycolysis and pentose phosphate pathway (PPP), were suppressed by ML-ESPs. Interestingly, tricarboxylic acid cycle (TCA)-related genes, such as pyruvate dehydrogenase phosphatase 1 (PDP1), PDP2, aconitate hydratase 1 (ACO1) and oxoglutarate dehydrogenase (OGDH) were upregulated by ML-ESPs. In summary, the transcriptome profiling of A549 cells were significantly altered by ML-ESPs. And we also provide new insight into how ML-ESPs induced a transcriptional reprogramming of glucose metabolism-related genes in A549 cells.

## 1. Introduction

*Trichinella spiralis* (*T. spiralis*) secretes multiple products during different niches, such as the muscle larva excretory/secretory products (ML-ESPs). ML-ESPs have been reported to have immunomodulatory potential and antitumor value (1). ML-ESPs can affect the proliferation, migration, and immunity of cancer cells *in vitro*, as well as tumorigenesis and metastasis *in vivo* (2–5). Previous research reports stated that ML-ESPs trigger apoptosis and S-phase arrest to inhibiting the proliferation of the A549 non-small cell lung cancer (NSCLC) cell line (6). However, the role played by ML-ESPs in the transcriptional regulation of A549 cells remains largely unknown. Determining the various effects of ML-ESPs in A549 cells at the transcriptome level is very important to more extensively understand the molecular function and anti-NSCLC mechanism of ML-ESPs and cite scientific support for their usage in the creation of potent antitumor therapies.

Lung cancer is the malignancy with highest mortality rate and incidence rate, according to the recent global cancer statistical report (7). There are two primary types of lung cancer: small cell lung cancer (SCLC) and NSCLC, with NSCLC making up more than 80% of instances of lung cancer (8). In recent years, the clinical treatment of NSCLC has been improving and the relative survival rate of NSCLC patients has increased. However, the metastasis of cancer cells, damage to normal cells, toxicity and resistance to therapeutic drugs acquired during the course of disease treatment remain unsatisfactory (9). In order to increase the efficacy of NSCLC treatment, it is crucial to apply biological reagents and identify therapeutic targets genes.

The technologies of RNA sequencing (RNA-seq) and microarray offer a variety of potential methods to transcriptomics. Growing evidence reveals the potential applications of transcriptomics in understanding how dysregulated genes affect the interactions between parasites and their hosts (10, 11). RNA-seq technique revealed novel genes involved in host parasite interactions and stage-specific expression patterns of miRNAs in *T. spiralis’* various developmental phases (12, 13). The expression changes in muscle cell proliferation, apoptosis and signaling pathways in response to *T. spiralis* were analyzed by microarray (10). ML-ESPs seemingly increase the transcriptomic diversity in cells. Therefore, studying the early change induced in A549 cells treated with ML-ESPs is expected to provide evidence for a improved comprehension of the anti-NSCLC mechanism of *T. spiralis*.

The transcriptome profile of genes expression and alternative splicing in A549 cells treated with ML-ESPs were thoroughly examined in this study. Differentially expressed genes (DEGs), novel genes, genes interaction networks and alternative splicing events (ASEs) were identified in A549 cells treated with ML-ESPs, which increased the diversity of the A549 cells transcriptome. In addition, bioinformatics analysis revealed that numerous DEGs were enriched to metabolic processes. What’s more, we found that ML-ESPs showed the potential to reprogram the expression of glucose metabolism-related genes in A549 cells. These results provide unique insight into the global profiling of ML-ESPs mediated transcriptional reprogramming of A549 cells.

## 2. Materials and methods

### 2.1. Animals

Specific pathogen free female Kunming mice (6 ~ 8 weeks old) were purchased from Liaoning Changsheng Biotechnology Co., Ltd. and maintained in a controlled environment (a 12/12 h light/dark cycle with a humidity of 55% and a temperature of 22 ± 2°C). The study was authorized by the Animal Care and Use Committee of Chengde Medical University (CDMULAC-20191202-013), and all operations involving animals were carried out strictly in line with the Chinese National Institute of Health Guide for the Care and Use of Laboratory Animals.

### 2.2. Preparation of ML-ESPs

Mice infected for 35 days with *T. spiralis*, 300 *T. spiralis* were administered orally, were cervically dislocated and executed. All of the mice’s striated muscle should be placed in a blender to be crushed. Hydrochloric acid and pepsin should then be added. The mixture should be digested for 3–4 h at 37°C in a water bath before the meat dregs and fibers are removed. Three repetitions of thorough washing with stroke-physiological saline solution containing 500 U mL^−1^ mycillin were performed on the *T. spiralis* ML, filter and precipitate with 100 mesh sterile copper mesh to collect pure muscle larvae. Then transferring them at a density of 6,000 larvae mL^−1^ to Roswell Park Memorial Institute (RPMI) 1,640 (Gibco, USA) medium containing 500 U mL^−1^ mycillin. The medium containing larvae was then put into an incubator with 5% CO_2_ and maintained at a constant 37°C temperature for 24 h. The supernatant was obtained after centrifuging the culture fluid at 12,000 × *g* for 30 min at 4°C. The 0.22 μm filter paper was used to filter the ML-ESPs, to obtain sterile ML-ESPs. Measure the proteins concentration using Bicinchoninic Acid Assay (BCA) Protein Assay Kit (Solarbio, China) and store it in a refrigerator at −80°C.

### 2.3. A549 cell culture

Complete RPMI 1640 (with 10% bovine calf serum and 1% 100 × penicillin–streptomycin (Solarbio, China)) were used to culture A549 cells, and the culture medium was replaced every 24 h in an incubator at 37°C with 5% CO_2_. When reaching 70 ~ 80% confluence, A549 cells were treated with ML-ESPs diluted with serum free 1,640 culture medium at a final concentration of 1.20 mg mL^−1^ for 24 h, and these cells constituted the ML-ESPs group. Other A549 cells were cultured in complete RPMI 1640 medium and composed the normal control (NC) group cell.

### 2.4. Library construction and RNA-seq

Total RNA was isolated from the ML-ESPs group and NC group using TRIzol (Invitrogen, USA). A NanoDrop ND-1000 spectrophotometer (NanoDrop Technologies, USA) was used to measure the concentration of total RNA and to evaluate the quality of the total RNA using agarose gel electrophoresis. The NEBNext^®^ Poly (A) mRNA Magnetic Isolation Module (New England Biolabs, USA) were used to enrich mRNA using a total of 2 μg of total RNA per sample, and the RiboZero Magnetic Gold Kit (Illumina, USA) was used to remove rRNA. KAPA Stranded RNA-Seq Library Prep Kit (Illumina, USA) was used to create a new RNA (processed) library, and Agilent 2100 Bioanalyzer (Agilent Technologies, USA) was used to evaluate the new RNA library’ quality. The new RNA library was quantified using quantitative PCR. Utilizing an Illumina NovaSeq 6000 (Illumina, USA), high-throughput sequencing was carried out.

### 2.5. Data processing

FastQC (Quality control) was used to verify the raw sequencing data produced by the Illumina NovaSeq 6000. Trimmed data was created by using Cutadapt to eliminate low-quality reads, 3′-adaptor sequences and 5′-adaptor sequences. *Q* = −log10 (*P*) was used to calculate each sample’s Q30 (error rate ≤ 0.1%), and a Q30 score greater than 80% indicated high-quality sequencing data. HISAT2 was used to independently map the newly obtained read pairs to the reference genome transcriptome (14). Information on the mapped, mtRNA, rRNA, and unmapped sequences was gathered.

### 2.6. Differentially expressed genes and transcript filtering

The Ballgown R package was employed to calculate the per kilobase of a gene or transcript per million mapped fragments (FPKM) ratio (15, 16). DEGs and differentially expressed transcripts (DETs) between the ML-ESPs group and the NC group were discovered according to the following criteria: a threshold Fold Change (FC) =1.5, *p* < 0.05 and a mean FPKM ≥0.5.

### 2.7. Novel gene and transcript identification

The sequencing data and genome were compared by HISAT2, and the abundance of the transcripts were determined with StringTie based on the comparison of the results from each sample (17). The assembly results of all samples were combined using StringTie, and the structures of genes and transcripts were merged and optimized by combining the annotation information. Gene-level and transcript-level expression calculations were performed with Ballgown, and these data were screened to identify novel gene and transcripts. Transcripts and novel genes were identified according to FPKM using R package Ballgown calculations with a FPKM ≥0.5 threshold. The coding potential assessment tool (CPAT) was used to evaluate the new genes’ potential to code for proteins (18).

### 2.8. ASEs identification

Five types of ASEs were identified by rMATS (19), comprising retained introns (RIs), mutually exclusive exons (MXEs), alternative 3′ splice sites (A3SSs) alternative 5′ splice sites (A5SSs), and skipped exons (SEs).

### 2.9. Bioinformatics analysis

The functions of the DEGs were identified using the Gene Ontology (GO)^1^ and Kyoto Encyclopedia of Genes and Genomes (KEGG)^2^ databases. Based on the interactions between the *Homo sapiens* genes in the STRING database,^3^ a gene interaction network was created using Cytoscape (version 3.9.0). The relationship between different nodes was calculated based on the betweenness centrality algorithm, which is used as the evaluation standard for genes interaction network. Betweenness centrality was calculated using cytoCNA in Cytoscape.

### 2.10. Quantitative real-time polymerase chain reaction (qRT-PCR)

Glycolysis-associated genes, including phosphofructokinase muscle (PFKM), phosphofructokinase liver (PFKL), enolase 2 (ENO2), lactate dehydrogenase B (LDHB); TCA-related genes, including pyruvate dehydrogenase phosphatase 1 (PDP1), PDP2, aconitate hydratase 1 (ACO1) and oxoglutarate dehydrogenase (OGDH); and PPP-related genes, including 6-phosphogluconolactonase (6PGL), ribulose-phosphate-3-epimerase (PRE), transketolase (TKT), transaldolase 1 (TALDO1), were determined through qRT-PCR. All the primers used in this study had been previously published in reference articles (20–22). First Strand cDNA Synthesis Kit (Beyotime, Beijing) was used according to the manifactirer’s instruction. Incubate at 42°C for 60 min to obtain cDNA. A SYBR Green Real-Time PCR Master Mix Kit (TransGen Biotech, Beijing) was used according to the manufacturer’s instructions. Initial denaturation at 95°C for 30 s, then followed by 45 cycles of 95°C for 5 s, 60°C for 60 s and 72°C for 30 s. The relative FCs of target genes expression were quantified with β-actin used as the endogenous reference genes and the 2^−ΔΔCt^ formula.

### 2.11. Statistical analysis

Software SPSS v22.0 (IBM Corp., USA) was used to conduct the data analysis. Independent samples *t*-test was used to analyze differences between two groups. The results were expressed as mean ± standard deviation. *P* < 0.05 was considered statistically significant.

## 3. Results

### 3.1. ML-ESPs significantly altered the transcriptomic landscape of A549 cells

**Figure 1:**
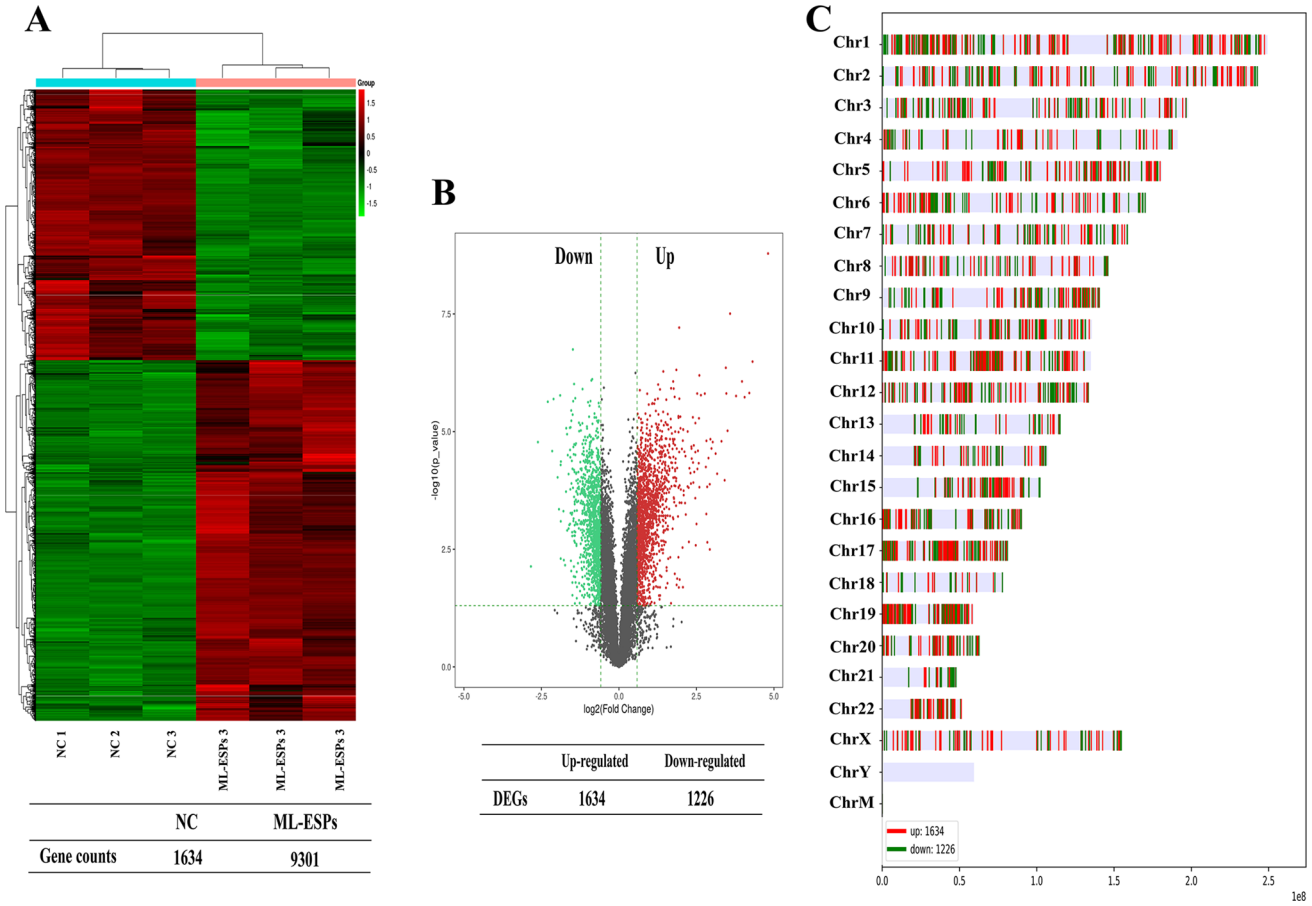
Overview of the transcriptional profile of A549 cells treated with ML-ESPs. Heatmap displaying transcriptional changes in the ML-ESPs group compared with the NC group **(A)**. Volcano plot visualizing the DEGs identified in the ML-ESPs group **(B)**. Detailed chromosomal locations of the DEGs **(C)**.

To investigate the transcriptional regulation by which ML-ESPs alter genes expression in A549 cells, we performed an extensive analysis of the RNA-seq dataset. Each sample was analyzed in three independent replicates. A total of 11,553 genes and 27,342 transcripts were identified in the NC group, while 11,877 genes and 27,871 transcripts were identified in the ML-ESPs group. The expression levels of all the detected genes from the ML-ESPs and NC samples were quantified using Ballgown. A hierarchical clustering heatmap analysis of the DEGs expression levels showed significant differences between the ML-ESPs group and NC group (Figure 1A). We used strict criteria, FC ≥ 1.5, *p* < 0.05 and mean FPKM ≥0.05, to identify DEGs. A total of 2,860 DEGs (1,634 DEGs were upregulated and 1,226 DEGs were downregulated) were identified. Volcano plot of all expressed genes displayed that ML-ESPs induced DEGs in A549 cells (Figure 1B). The DEGs were distributed on all chromosomes except chromosomes Y. Chromosomes 1, 11, and 19 showed a higher number of DEGs than the other chromosomes (Figure 1C). These findings demonstrated that ML-ESPs have the capacity to significantly influence the expression of genes and chromosome distribution in A549 cells.

### 3.2. Bioinformatic analysis of the DEGs in A549 cells treated with ML-ESPs

**Figure 2:**
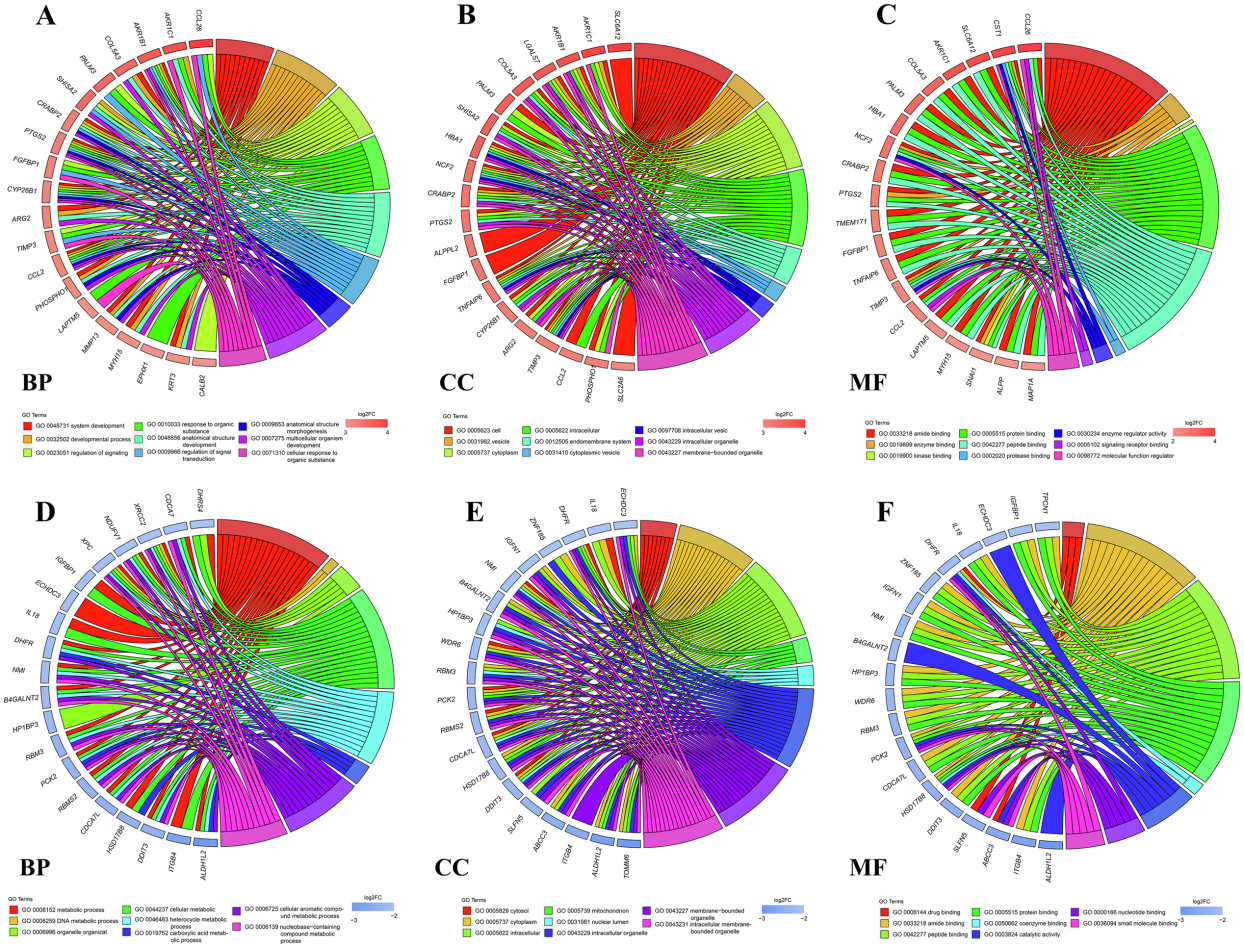
GO function annotation of enriched DEGs. GO chord plot showing upregulated DEGs **(A)** and downregulated DEGs **(D)** and the corresponding GO terms in the biological process (BP) category. GO chord plot showing upregulated DEGs **(B)** and downregulated DEGs **(E)** and the corresponding GO terms in the cellular component (CC) category. GO chord plot showing upregulated DEGs **(C)** and downregulated DEGs **(F)** and the corresponding GO terms into the molecular function (MF) category. The genes are linked to their GO terms via colored ribbons. Genes are ordered according to the observed log2-fold change (log2FC), which is displayed in red squares with increasing intensity or blue squares with decreasing intensity next to the selected genes.

To decipher the probable biological roles of these DEGs, we carried out GO and KEGG enrichment analysis of both upregulated and downregulated DEGs. The GO biological process (BP) terms enriched with upregulated DEGs were closely related to the regulation of signaling and response to organic substance (Figure 2A). The GO BP terms enriched with downregulated DEGs were associated with DNA replication and metabolic processes (Figure 2D). The upregulated DEGs enriched in GO cellular component (CC) terms were mainly in the endomembrane system, cytoplasm and vesicle (Figure 2B); the downregulated DEGs in GO CC terms were mainly enriched in intracellular cells and mitochondrion (Figure 2E). The upregulated DEGs in GO molecular function (MF) terms mainly played protein binding, signaling receptor binding functions (Figure 2C), and the downregulated DEGs enriched in GO MF terms mainly performed ligase activity and catalytic activity (Figure 2F).

**Figure 3:**
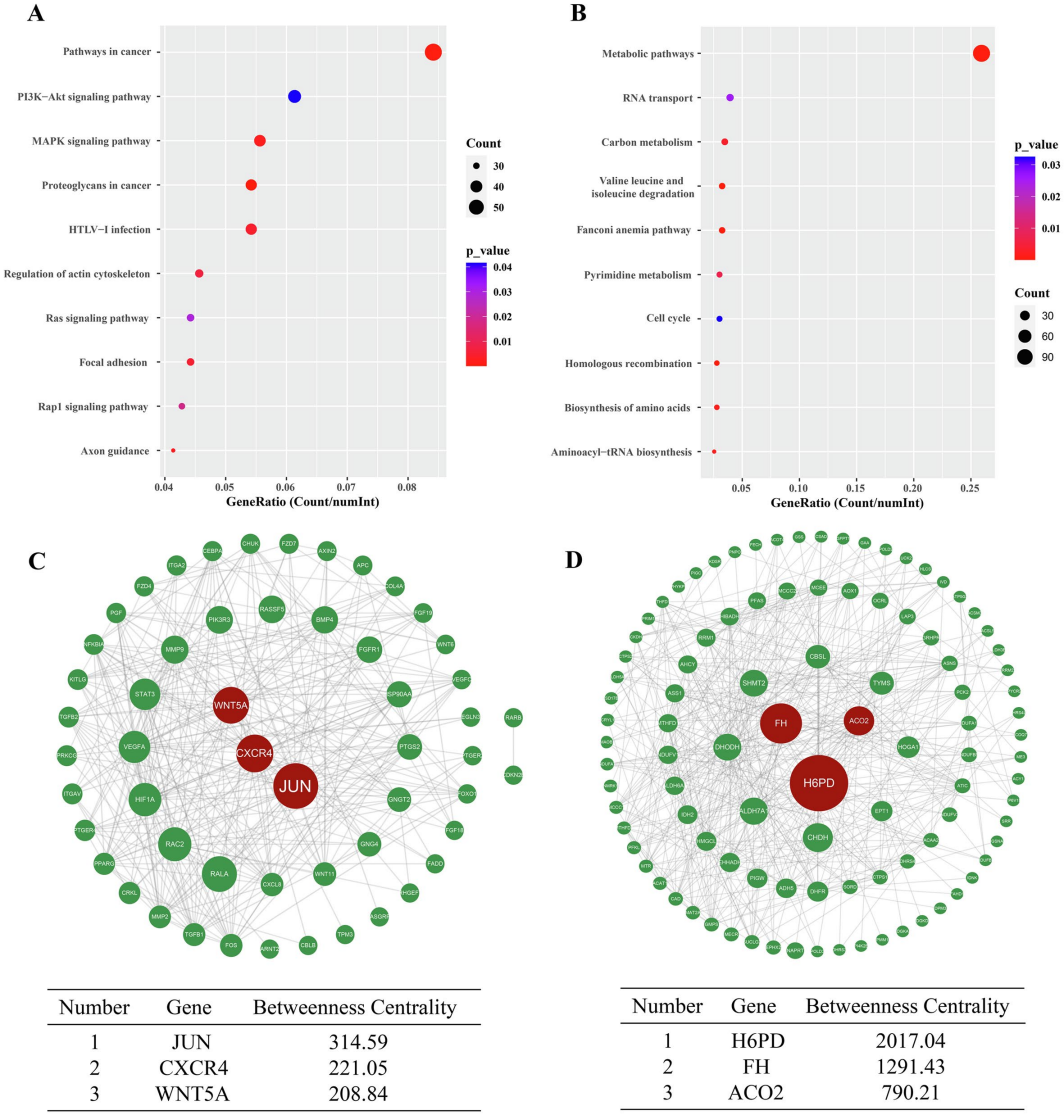
Bubble diagram of the 10 KEGG pathways most enriched with DEGs. The 10 KEGG pathways with the highest number of upregulated DEGs **(A)**. The 10 KEGG pathways with the highest number of downregulated DEGs **(B)**. Genes interactions network of DEGs enriched in “Pathways in cancer” term based on betweenness centrality algorithm **(C)**. Genes interactions network of DEGs in “Metabolic pathways” term based on betweenness centrality algorithm **(D)**.

A total of 112 entries were enriched in KEGG analysis. The 10 pathways with the most enriched genes were visualized. The upregulated DEGs were mainly related to signaling pathways affecting tumor cell development, such as MAPK signaling pathway, PI3K/AKT signaling pathway and Ras signaling pathway (Figure 3A). The downregulated DEGs were mainly related to metabolism, such as metabolic process and carbon metabolism (Figure 3B). Then, the genes interaction networks of the DEGs in the pathways in cancer term and metabolic processes term were plotted. JUN, CXCR4, and WNT5A were the top three interaction DEGs in pathways in cancer (Figure 3C); H6PD, FH, and ACO2 (Figure 3D), which associate with glucose metabolism, were the top three interaction DEGS in metabolic processes. GO and KEGG enrichment analysis indicated the major changed biological process inA549 cells. What’s more, genes interaction networks of metabolic processes pointed to glucose metabolism biological process.

### 3.3. Novel genes and transcripts were identified in A549 cells treated with ML-ESPs

**Figure 4:**
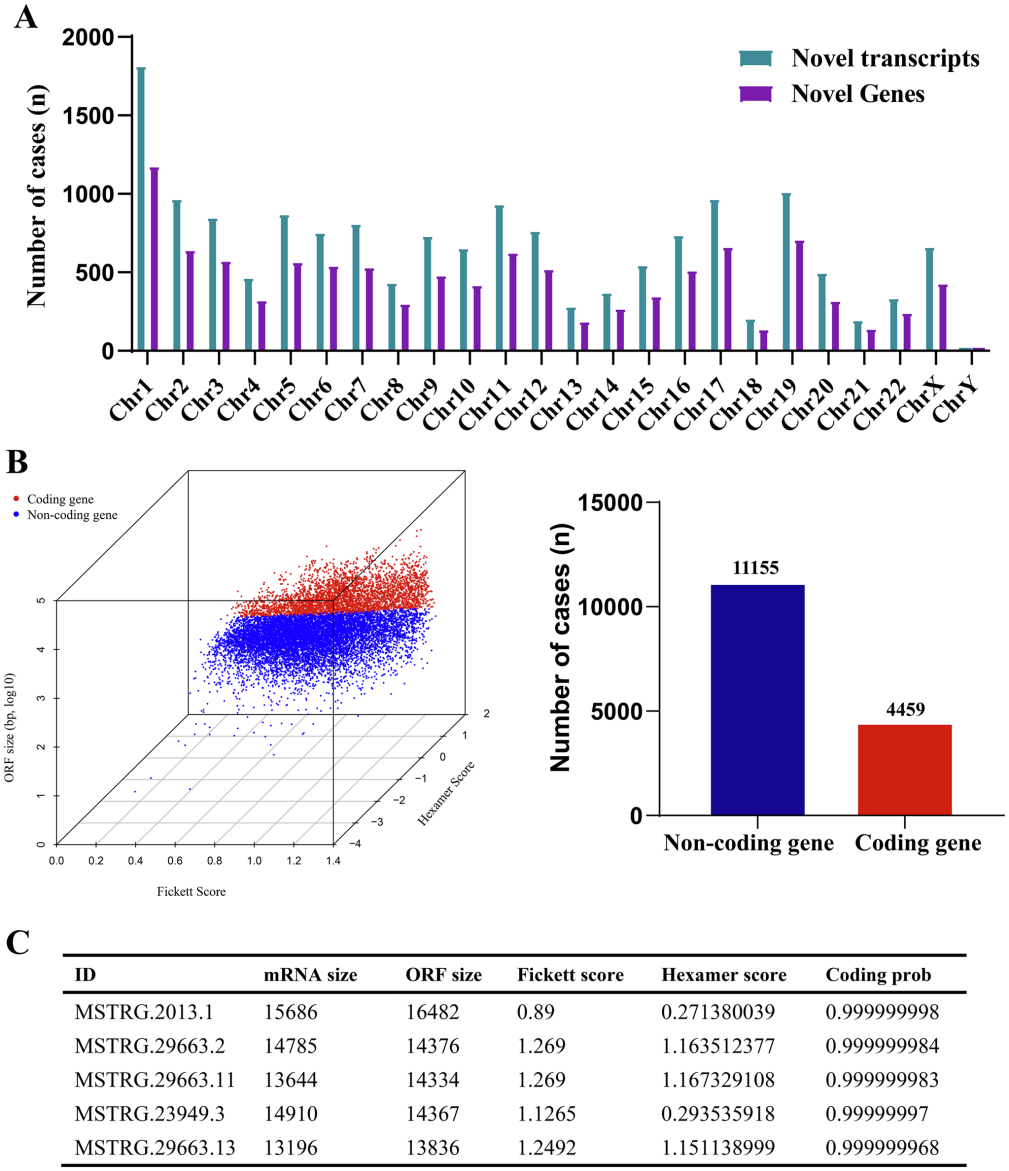
Overview of novel genes. Chromosomal locations of novel genes and novel transcripts **(A)**. Coding probability of novel genes **(B)**. Details of the five novel genes most enriched among the novel coding genes **(C)**.

In A549 cells, ML-ESPs can either stimulate or inhibit a variety of biological processes. To research the transcripts and novel genes involved in these processes, we identified novel genes and transcripts, and the coding ability of the identified transcripts was evaluated based on open reading frame (ORF) size, hexamer complex and so on. Overall total of 10,364 novel genes and 15,614 novel transcripts were discovered in A549 cells treated with ML-ESPs. The chromosome locations of novel genes and transcripts showed chromosome 1 contained the largest number of novel genes (1161) and novel transcripts (1799) (Figure 4A). The coding ability of the novel genes was assessed according to ORF size, hexamer complex use preference score and Fickett score. According to the results, only 4,459 novel genes were found to have the coding potential (Figure 4B). MSTRG.2013.1, MSTRG.29663.2, MSTRG.29663.11, MSTRG.23949.3, and MSTRG.29663.13 were the 5 most abundant novel transcripts ID with coding potential (Figure 4C). Identification of novel genes and novel transcripts with coding capacity showed enrichment in the transcriptional regulation of ML-ESPs levels in A549 cells.

### 3.4. ML-ESPs induced different ASEs in A549 cells

**Figure 5:**
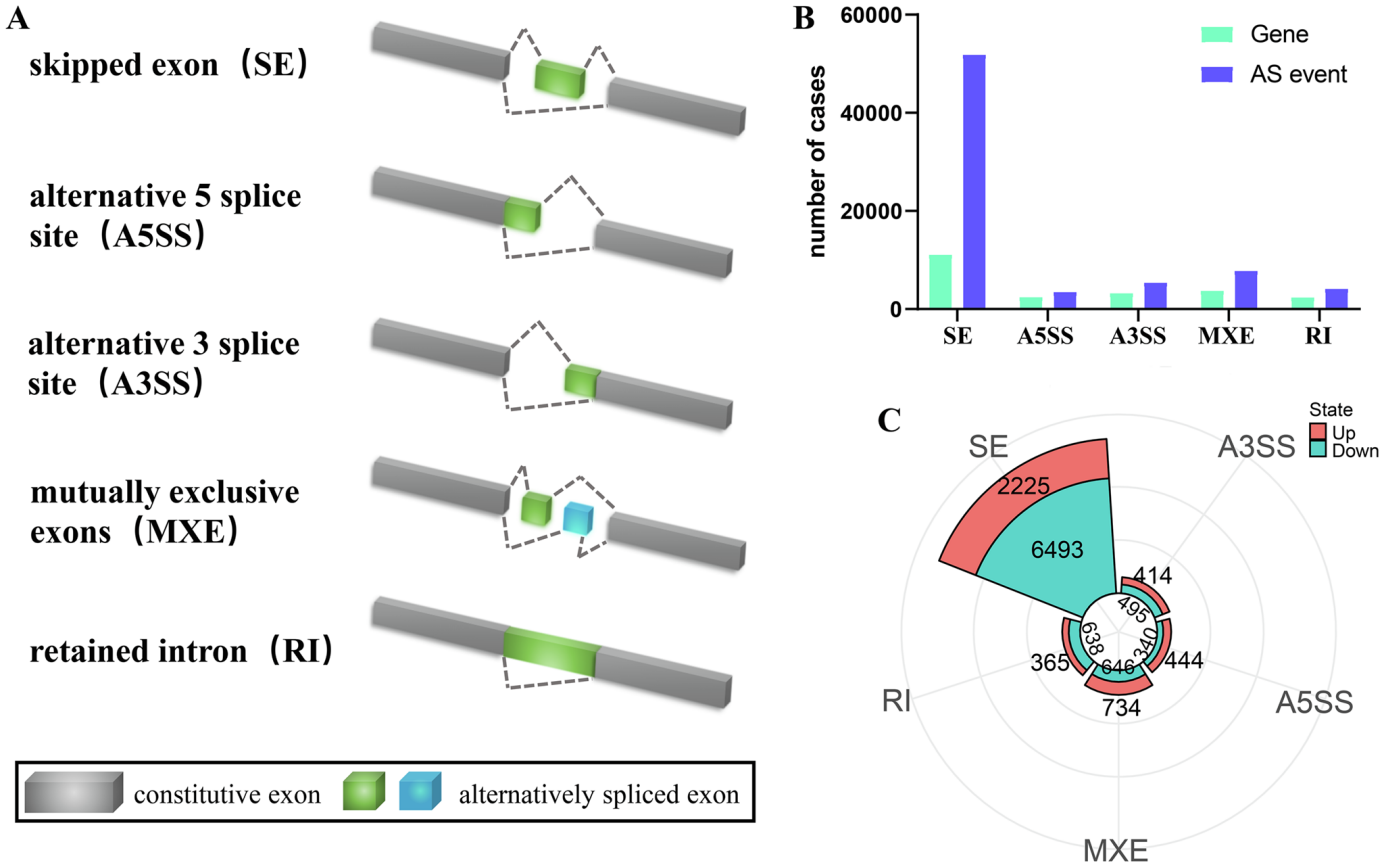
Overview of ASEs. Illustrations of the five types of AS events: SEs, A5SSs, A3SSs, MXEs, and RIs **(A)**. A number of AS events and corresponding genes in the ML-ESPs group **(B)**. The five most prevalent ASEs in downregulated and upregulated **(C)**.

One definitive aim of this study was acquiring gain insight into how the alternative splicing regulatory role played by ML-ESPs. SE, A5SS, A3SS, MXE, and RI were the 5 splicing patterns identified (Figure 5A). A total of 72,784 ASEs were found in 23,078 genes, including 51,892 SEs in 11,142 genes, 3,500 A5SSs in 2,457 genes, 5,413 A3SSs in 3,293 genes, 7,806 MXEs in 3,754 genes, and 4,173 RIs in 2,432 genes (Figure 5B). Among the differentially expressed ASEs, 8,718 SEs (in 2,225 upregulated and 6,493 downregulated genes), 784 A5SSs (in 444 upregulated and 340 downregulated), 909 A3SSs (in 414 upregulated and 495 downregulated), 1,380 MXEs (in 734 upregulated and 646 downregulated), and 1,003 RIs (in 365 were upregulated and 638 downregulated) (Figure 5C). ASEs, generating different transcripts by splicing genes, increased the diversity of the transcriptome in A549 cells in response to ML-ESPs.

### 3.5. ML-ESPs induced a transcriptional reprogramming of glucose metabolism-related genes in A549 cells

**Figure 6:**
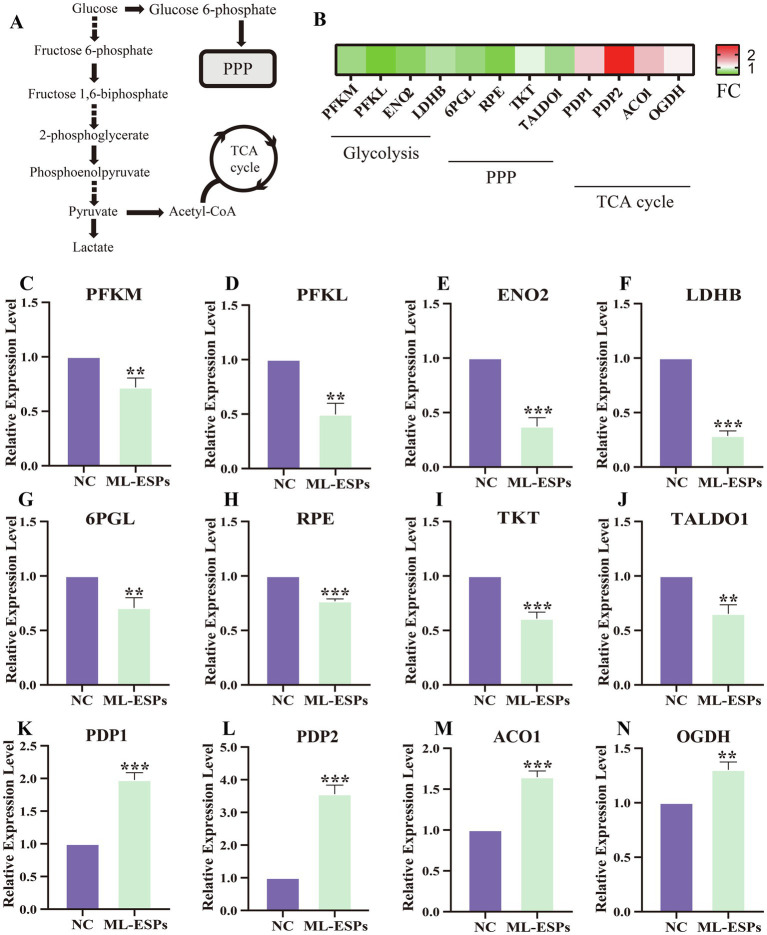
Alterations of glycolysis-, PPP- and TCA cycle-related genes. A schematic diagram of glycolysis, PPP and TCA cycle **(A)**. Visualization based on FC in glycolysis-, PPP- and TCA cycle-related genes **(B)**. A549 cells were treated with ML-ESPs for 24 h, and mRNA levels were analyzed by qRT-PCR **(C–N)**. Results are presented as the means ± SDs from three independent experiments. *p < 0.05, **p < 0.01, ***p < 0.001, compared with the NC group.

The genes interactions network of the downregulated DEGs indicated that there are significant changes in the genes related to the glucose metabolism process. We then focused our attention on the glycolysis, PPP and TCA cycle in A549 cells (Figure 6A). Heatmap of glycolytic, PPP and TCA cycle-related genes based on FC are shown (Figure 6B). To confirm the early effect of ML-ESPs on glycolytic, PPP and TCA cycle in A549 cells, we detected the mRNA expression of glycolytic-, PPP- and TCA cycle-related genes by qRT-PCR. The two isoforms of PFK, which is the rate limiting enzyme in glycolysis, are PFKM and PFKL, both of which were significantly lower in the ML-ESPs groups than in the NC group (Figures 6C,D). The ENO2 mRNA level was lower in the ML-ESPs compared to NC group (Figure 6E). The LDHB mRNA were significant downregulated by ML-ESPs (Figure 6F). PPP-related genes, such as 6PGL, RPE, TKT and TALDO1, were also significant downregulated in response to ML-ESPs in A549 cells (Figures 6G–J). However, the level of two isoforms of PDP mRNA, which encode enzymes critical for connecting glycolysis and with TCA cycle, were PDP1 and PDP2, both of which were significantly higher in ML-ESPs compared to NC group (Figures 6K,L). The expression both of ACO1 and OGDH mRNA, encoding enzymes important to TCA cycle, were also upregulated by ML-ESPs (Figures 6M,N). These results demonstrated that ML-ESPs reprogramed the expression of glycolysis-, PPP- and TCA cycle-related genes.

## 4. Discussion

*Trichinella spiralis*, which is among of the helminth zoonoses most frequently transmitted through food, causes a globalized zoonotic parasitic disease known as trichinosis. Interestingly, ML-ESPs show the capacity to inhibit tumor proliferation by triggering apoptosis pathway or regulate the cellular immune system by regulating interleckin-10 (23). At the early stage of *T. spiralis* infection, liver cancer cell apoptosis was induced by significantly increasing the levels of Th1 cytokines (3). ML-ESPs show the capacity to arrest cells in the S-phase (6). By activating genes associated with the mitochondrial pathway, including Bax, Cytochrome-c, caspase-3, and caspase-9, ML-ESPs induced apoptosis of H446 SCLC cells line (5). These characteristics suggest that *T. spiralis* may exhibit an antitumor mechanism. However, the involvement of ML-ESPs in lung cancer progression, especially its impact on transcriptional and post-transcriptional regulation of NSCLC, has not been fully elucidated.

To identify the cellular responses and effects of low or moderate expressed transcribed genes, we systematically studied the transcriptomic diversity in A549 cells treated with ML-ESPs at an early phase of treatment using RNA-seq. The RNA-seq data showed a total of 2,860 DEGs were identified. GO analysis revealed that up-regulated DEGs were primarily related with response to organic substance and down-regulated DEGs were primarily related with metabolic process. KEGG analysis revealed that up-regulated DEGs were mainly related with pathway in cancer. Parasites regulate tumor progression through different cancer signaling pathways, such as MAPK, JAK–STAT, Wnt and PI3K-Akt signaling pathways (10) and PI3K was up-regulated in infected muscle cells during *T. spiralis* infection (24). However, the down-regulated DEGs were primarily related with metabolic process, which suggests that the metabolic process of A549 cells may change under the action of ML-ESPs. For example, it has been shown that *T. spiralis* derived antigens alleviate obesity in mice; *Microplitis manila* changes host metabolism and others (25, 26). We further conducted genes interactions network analysis on DEGs in metabolic process. Among the down-regulated DEGs in metabolic process, the most significant interaction factor is H6PD FH ACO2. H6PD has a hexose-6-phosphate dehydrogenase activity and catalyzes the first step of the pentose phosphate pathway (27, 28). Inhibition of H6PD expression levels produces suppressive effects on pancreatic cancer cells (29) H6PD overexpression locally offset the effects of TUG1 deficiency on retinoblastoma proliferation and apoptosis (30). Aconitase plays a role in regulating resistance to oxidative stress and cell death (31). ACO2 may be used as an immunotherapeutic and potential prognostic biomarker for several cancers, including hepatocellular carcinoma (32). ACO2 correlates with colorectal cancer cell survival (33). Metabolic reprogramming occurs in FH deficient-cells (34) Patients with low FH expression exhibit poorer overall survival (35). The results of genes interaction network suggested that the key interacting genes in the metabolic process were involved in the glucose metabolism of cells. Glycometabolism is the basis for cell proliferation, and cancer cells in particular, are more energy dependent because of rapid cell proliferation. Parasites play a variety of roles in regulating glucose metabolism. Research shows that *Trypanosoma cruzi* promotes glycolysis activation with HIF-1α-dependent in cardiomyocyte (36); *Leishmania* infantum Infected dendritic cells change the expression of primarily genes related to metabolism and particularly glycolysis (37).

To our knowledge, our study is the first to systematically identify and examine novel genes and ASEs in A549 cells treated with ML-ESPs. Overall total of 15,614 novel transcripts and 10,364 novel genes were discovered, and only 4,459 of the novel genes presented the ability to code. The ASEs of A549 cells caused by ML-ESPs are still a mystery. A few studies have examined the ASEs in cells that had been exposed to parasites, such as *Toxoplasma gondii* rhoptry protein 18 (38), rhoptry organelle protein 17 (39) and *Trypanosoma cruzi* (40). By contrasting five splicing products, including SE, A5SS, A3SS, MXE, and RI, we looked into the function of ML-ESPs in the regulation of A549 cellular ASEs. For example, SE, a form of ASEs, is a biomarker that can be used to treat non-small-cell lung cancer such as MET exon 14 (41). Another type of ASEs that can introduce ID elements to the protein is RI (42) or causes mRNA degradation through RNA monitoring mechanism (43). The exact mechanism of ML-ESPs changing SE and RI events is still ambiguous. Further experiments, such as analyzing the components of ML-ESPs by mass spectrometry, screening a single protein, and transfecting each gene into A549 cells independently to determine the role of ASEs, will further clarify the mechanism and operation of ML-ESPs processing to induce ASEs.

Then, we turn our attention to dysregulated genes associated with glucose metabolism. We found that ML-ESPs repressed the expression of mRNAs related to glycolysis and PPP, including PFKM, PFKL, ENO2, LDHB, 6PGL, RPE, TKT, and TALDO1, but activated the expression of mRNAs within the TCA cycle, including PDP1, PDP2, ACO1, and OGDH. These results suggest that ML-ESPs show the potential to switch A549 cells from undergoing cytosolic aerobic glycolysis to undergoing the mitochondrial TCA cycle. PFKM is the rate limiting enzyme of glycolysis, affecting the rate and likelihood for glycolysis in cells. PFKM expression levels have also been associated with cancer development and metastasis (44), LDHB catalyzes the interconversion of pyruvate and lactate. High LDHB expression has been significantly correlated with serum LDH concentration and clinical stage of NSCLC (45, 46). Recent research demonstrated that LDHB silencing decreased the development and spread of xenograft tumors (47), TKT, a crucial enzyme operating between the oxidative arm and the nonoxidative arm of PPP, is highly expressed in the tissues of NSCLC patients (48). Inhibition of TKT expression promotes drug sensitivity to gefitinib in NSCLC (49). PDP1 catalyzes dephosphorylation of pyruvate dehydrogenase complex E1 while reactivating the alpha subunit. PDP1 plays an significant role in the invasion and spread of malignant cell (50). These genes are correlated with the occurrence and development of NSCLC. The finding that ML-ESPs controlled the mRNA expression of their downstream targets suggested that ML-ESPs might influence the progression of NSCLC by controlling the expression of these genes. To pinpoint the precise mechanism by which ML-ESPs mediate biological processes, more research must be done. For instance, western blot analysis can be performed to identify changes in the protein levels of the above-mentioned genes at the cell level.

ML-ESPs induced a transcriptional reprogramming of glucose metabolism-related genes in A549 cells. In particular, ML-ESPs downregulated the glycolysis and PPP-related genes, but upregulated the TCA-cycle associated genes. According to Warburg effect, cancer cells preferentially undergo glycolysis even under conditions of adequate oxygen (51). What’s more, a high rate of glycolysis, which then meets the energy required for cell proliferation, leads to more intracellular production and increased secretion of lactic acid. This lactate secretion creates an increasingly acidic microenvironment for the cell, which facilitates invasion and metastasis. Affecting glycolysis is thought to be an effective tactic to reduce metastasis in cancer (52–54). Therefore, the down-regulation of glycolysis-related genes by ML-ESPs may be detrimental to A549 cells. In fact, it has been documented that ML-ESPs inhibit the proliferation of tumor cells (5, 6). By the way, the up-regulation of TCA-cycle associated genes may be a compensatory phenomenon. It has been shown that TCA cycle related gene defected-cells increase their glycolytic rates and instead of oxidizing glucose in the mitochondria they shunt it into lactate production (55).

## 5. Conclusion

This study presents the first RNA-Seq based analysis of the transcriptomic responses of A549 cells to ML-ESPs. 2,860 genes were significantly altered by ML-ESPs, including 1,634 up-regulated genes and 1,226 down-regulated genes. The functions of significantly altered down-regulated genes were mainly involved in metabolic process. And, the expression of glycolysis-, PPP- and TCA cycle-related genes were changed to different degrees in A549 cells treated with ML-ESPs. Our data revealed several potential new roles of ML-ESPs in the transcriptional regulation of A549 cells. Additionally, five kinds of ASEs increase transcriptomic diversity by generating different transcripts by splicing genes. These results provide novel insights into understanding the molecular functions and potential regulatory mechanisms of ML-ESPs in A549 cells.

## Data availability statement

The datasets presented in this study can be found in online repositories. The names of the repository/repositories and accession number(s) can be found at: https://www.ncbi.nlm.nih.gov/bioproject/; PRJNA967794.

## Ethics statement

The animal study was reviewed and approved by the Animal Care and Use Committee of Chengde Medical University (CDMULAC-201991202-013).

## Author contributions

HW: data curation, investigation, writing—original draft, and formal analysis. YZ and ML: data curation and investigation. JP, DL, and W-PG: methodology and validation. GX: conceptualization and writing—review and editing. LD: conceptualization, funding acquisition, supervision, and writing—review and editing. All authors contributed to the article and approved the submitted version.

## Funding

This study was supported by Hebei Provincial Science and Technology Department’s “special project for guiding technological innovation – consultation on scientific and technological work” project (No. KY202002), Key disciplines in colleges and universities in Hebei Province (Ji Jiao Gao [2013] No. 4), and Project cultivation fund of National Natural Science Foundation of China (202005).

## Conflict of interest

The authors declare that the research was conducted in the absence of any commercial or financial relationships that could be construed as a potential conflict of interest.

## Publisher’s note

All claims expressed in this article are solely those of the authors and do not necessarily represent those of their affiliated organizations, or those of the publisher, the editors and the reviewers. Any product that may be evaluated in this article, or claim that may be made by its manufacturer, is not guaranteed or endorsed by the publisher.

## Footnotes

^1^ http://www.geneontology.org/

^2^ https://www.kegg.jp/

^3^ https://string-db.org/

## References

[1] Ilic N Bojic-Trbojevic Z Lundstrom-Stadelmann B Cujic D Mitic I Gruden-Movsesijan A. Immunomodulatory components of *Trichinella spiralis* excretory-secretory products with lactose-binding specificity. EXCLI J. (2022) 21:793–813. doi: 10.17179/excli2022-4954 35949491 PMC9360477

[2] Elhasawy FA Ashour DS Elsaka AM Ismail HI. The apoptotic effect of *Trichinella spiralis* infection against experimentally induced hepatocellular carcinoma. Asian Pac J Cancer Prev. (2021) 22:935–46. doi: 10.31557/APJCP.2021.22.3.935 33773560 PMC8286675

[3] Ding J Tang B Liu X Bai X Wang Y Li S. Excretory-secretory product of *Trichinella spiralis* inhibits tumor cell growth by regulating the immune response and inducing apoptosis. Acta Trop. (2022) 225:106172. doi: 10.1016/j.actatropica.2021.106172, PMID: 34627760

[4] Han C Yu J Zhang Z Zhai P Zhang Y Meng S. Immunomodulatory effects of *Trichinella spiralis* excretory-secretory antigens on macrophages. Exp Parasitol. (2019) 196:68–72. doi: 10.1016/j.exppara.2018.10.001, PMID: 30316775

[5] Luo J Yu L Xie G Li D Su M Zhao X. Study on the mitochondrial apoptosis pathways of small cell lung cancer H446 cells induced by *Trichinella spiralis* muscle larvae ESPs. Parasitology. (2017) 144:793–800. doi: 10.1017/S0031182016002535, PMID: 28073393

[6] Wu H Li M Shao X An Z Du J Yin H. *Trichinella spiralis* muscle larvae excretory/secretory products trigger apoptosis and S-phase arrest of the non-small-cell lung cancer line A549. Exp Parasitol. (2020) 218:107983. doi: 10.1016/j.exppara.2020.107983, PMID: 32861680

[7] Siegel RL Miller KD Fuchs HE Jemal A. Cancer statistics, 2022. CA Cancer J Clin. (2022) 72:7–33. doi: 10.3322/caac.21708 35020204

[8] Sung H Ferlay J Siegel RL Laversanne M Soerjomataram I Jemal A. Global cancer statistics 2020: globocan estimates of incidence and mortality worldwide for 36 cancers in 185 countries. CA Cancer J Clin. (2021) 71:209–49. doi: 10.3322/caac.21660 33538338

[9] Miller M Hanna N. Advances in systemic therapy for non-small cell lung cancer. BMJ. (2021) 375:n2363. doi: 10.1136/bmj.n2363 34753715

[10] Hernandez-Ancheyta L Salinas-Tobon M Cifuentes-Goches JC Hernandez-Sanchez J. *Trichinella spiralis* muscle larvae excretory-secretory products induce changes in cytoskeletal and myogenic transcription factors in primary myoblast cultures. Int J Parasitol. (2018) 48:275–85. doi: 10.1016/j.ijpara.2017.10.002, PMID: 29258830

[11] Tebben K Dia A Serre D. Determination of the stage composition of *Plasmodium* infections from bulk gene expression data. mSystems. (2022) 7:e0025822. doi: 10.1128/msystems.00258-22, PMID: 35862820 PMC9426464

[12] Liu X Song Y Lu H Tang B Piao X Hou N. Transcriptome of small regulatory RNAs in the development of the zoonotic parasite *Trichinella spiralis*. PLoS One. (2011) 6:e26448. doi: 10.1371/journal.pone.0026448, PMID: 22096484 PMC3212509

[13] Liu X Song Y Jiang N Wang J Tang B Lu H. Global gene expression analysis of the zoonotic parasite *Trichinella spiralis* revealed novel genes in host parasite interaction. PLoS Negl Trop Dis. (2012) 6:e1794. doi: 10.1371/journal.pntd.0001794, PMID: 22953016 PMC3429391

[14] Kim D Langmead B Salzberg SL. HISAT: a fast spliced aligner with low memory requirements. Nat Methods. (2015) 12:357–60. doi: 10.1038/nmeth.3317, PMID: 25751142 PMC4655817

[15] Frazee AC Pertea G Jaffe AE Langmead B Salzberg SL Leek JT. Ballgown bridges the gap between transcriptome assembly and expression analysis. Nat Biotechnol. (2015) 33:243–6. doi: 10.1038/nbt.3172, PMID: 25748911 PMC4792117

[16] Pertea M Kim D Pertea GM Leek JT Salzberg SL. Transcript-level expression analysis of RNA-seq experiments with HISAT, StringTie and Ballgown. Nat Protoc. (2016) 11:1650–67. doi: 10.1038/nprot.2016.095, PMID: 27560171 PMC5032908

[17] Pertea M Pertea GM Antonescu CM Chang TC Mendell JT Salzberg SL. StringTie enables improved reconstruction of a transcriptome from RNA-seq reads. Nat Biotechnol. (2015) 33:290–5. doi: 10.1038/nbt.3122, PMID: 25690850 PMC4643835

[18] Wang L Park HJ Dasari S Wang S Kocher JP Li W. CPAT: coding-potential assessment tool using an alignment-free logistic regression model. Nucleic Acids Res. (2013) 41:e74. doi: 10.1093/nar/gkt006, PMID: 23335781 PMC3616698

[19] Shen S Park JW Lu ZX Lin L Henry MD Wu YN. rMATS: robust and flexible detection of differential alternative splicing from replicate RNA-Seq data. Proc Natl Acad Sci. (2014) 23:111, E5593–E5601. doi: 10.1073/pnas.1419161111 PMC4280593 25480548

[20] Zou Z Tsang JO Yan B Chik KK Chan CC Cao J. Metabolic profiling reveals significant perturbations of intracellular glucose homeostasis in enterovirus-infected cells. Meta. (2020) 10:302. doi: 10.3390/metabo10080302 PMC7466099 32717953

[21] Wu Z Jia J Xu X Xu M Peng G Ma J. Human herpesvirus 6A promotes glycolysis in infected T cells by activation of mTOR signaling. PLoS Pathog. (2020) 16:e1008568. doi: 10.1371/journal.ppat.1008568 32516328 PMC7282626

[22] Prusinkiewicz MA Tu J Dodge MJ MacNeil KM Radko-Juettner S Fonseca GJ. Differential effects of human adenovirus E1A protein isoforms on aerobic glycolysis in A549 human lung epithelial cells. Viruses. (2020) 12:610. doi: 10.3390/v12060610 32503156 PMC7354625

[23] Beiting DP Bliss SK Schlafer DH Roberts VL Appleton JA. Interleukin-10 limits local and body cavity inflammation during infection with muscle-stage Trichinella spiralis. Infect Immun. (2004) 72:3129–37. doi: 10.1128/IAI.72.6.3129-3137.2004 15155614 PMC415664

[24] Wu Z Nagano I Kajita K Nishina M Takahashi Y. Hypoglycaemia induced by *Trichinella* infection is due to the increase of glucose uptake in infected muscle cells. Int J Parasitol. (2009) 39:427–34. doi: 10.1016/j.ijpara.2008.09.001 18838075

[25] Tong M Yang X Liu H Ge H Huang G Kang X. The *Trichinella spiralis*-derived antigens alleviate HFD-induced obesity and inflammation in mice. Int Immunopharmacol. (2023) 117:109924. doi: 10.1016/j.intimp.2023.109924 36848791

[26] Gulinuer A Xing B Yang L. Host transcriptome analysis of *Spodoptera frugiperda* larvae parasitized by *Microplitis manilae*. Insects. (2023) 14:100. doi: 10.3390/insects14020100 36835669 PMC9966743

[27] Draper N Walker EA Bujalska IJ Tomlinson JW Chalder SM Arlt W. Mutations in the genes encoding 11beta-hydroxysteroid dehydrogenase type 1 and hexose-6-phosphate dehydrogenase interact to cause cortisone reductase deficiency. Nat Genet. (2003) 34:434–9. doi: 10.1038/ng1214, PMID: 12858176

[28] Lavery GG Walker EA Tiganescu A Ride JP Shackleton CH Tomlinson JW. Steroid biomarkers and genetic studies reveal inactivating mutations in hexose-6-phosphate dehydrogenase in patients with cortisone reductase deficiency. J Clin Endocrinol Metab. (2008) 93:3827–32. doi: 10.1210/jc.2008-0743, PMID: 18628520 PMC2579651

[29] Song Y Gao Z Zheng C. Silencing LINC01234 represses pancreatic cancer progression by inhibiting the malignant phenotypes of pancreatic cancer cells. Immunobiology. (2022) 227:152295. doi: 10.1016/j.imbio.2022.152295, PMID: 36343541

[30] Xiu C Song R Jiang J. TUG1 promotes retinoblastoma progression by sponging miR-516b-5p to upregulate H6PD expression. Transl Cancer Res. (2021) 10:738–47. doi: 10.21037/tcr-19-1480 35116405 PMC8799124

[31] Moeder W Del PO Navarre DA Martin GB Klessig DF. Aconitase plays a role in regulating resistance to oxidative stress and cell death in Arabidopsis and Nicotiana benthamiana. Plant Mol Biol. (2007) 63:273–87. doi: 10.1007/s11103-006-9087-x 17013749

[32] Wang Z Zheng W Chen Z Wu S Chang H Cai M. Pan-Cancer analysis shows that ACO2 is a potential prognostic and immunotherapeutic biomarker for multiple cancer types including hepatocellular carcinoma. Front Oncol. (2022) 12:1055376. doi: 10.3389/fonc.2022.1055376, PMID: 36531056 PMC9748622

[33] Zhang Z Zhu H Li Q Gao W Zang D Su W. Gene expression profiling of tricarboxylic acid cycle and one carbon metabolism related genes for prognostic risk signature of colon carcinoma. Front Genet. (2021) 12:647152. doi: 10.3389/fgene.2021.647152, PMID: 34589110 PMC8475515

[34] Carlo MI. Improving systemic therapy for fumarate hydratase-deficient renal cell carcinoma. Eur Urol. (2023) 83:e113–4. doi: 10.1016/j.eururo.2022.12.020 36682904 PMC13317412

[35] Vadhan A Yang YF Wang YM Chen PY Tzou SC Cheng KH. Fumarate hydratase inhibits non-small cell lung cancer metastasis via inactivation of AMPK and upregulation of DAB2. Oncol Lett. (2023) 25:42. doi: 10.3892/ol.2022.13627, PMID: 36589668 PMC9773317

[36] Venturini G Alvim JM Padilha K Toepfer CN Gorham JM Wasson LK. Cardiomyocyte infection by *Trypanosoma cruzi* promotes innate immune response and glycolysis activation. Front Cell Infect Microbiol. (2023) 13:1098457. doi: 10.3389/fcimb.2023.1098457, PMID: 36814444 PMC9940271

[37] Margaroni M Agallou M Vasilakaki A Karagkouni D Skoufos G Hatzigeorgiou AG. Transcriptional profiling of *Leishmania infantum* infected dendritic cells: insights into the role of immunometabolism in host-parasite interaction. Microorganisms. (2022) 10:1271. doi: 10.3390/microorganisms10071271 35888991 PMC9322131

[38] Li JX He JJ Elsheikha HM Ma J Xu XP Zhu XQ. ROP18-mediated transcriptional reprogramming of HEK293T cell reveals new roles of ROP18 in the interplay between *Toxoplasma gondii* and the host cell. Front Cell Infect Microbiol. (2020) 10:586946. doi: 10.3389/fcimb.2020.586946, PMID: 33330132 PMC7734210

[39] Li JX He JJ Elsheikha HM Chen D Zhai BT Zhu XQ. Toxoplasma gondii ROP17 inhibits the innate immune response of HEK293T cells to promote its survival. Parasitol Res. (2019) 118:783–92. doi: 10.1007/s00436-019-06215-y, PMID: 30675671

[40] Jung H Han S Lee Y. Transcriptome analysis of alternative splicing in the pathogen life cycle in human foreskin fibroblasts infected with *Trypanosoma cruzi*. Sci Rep. (2020) 10:17481. doi: 10.1038/s41598-020-74540-9, PMID: 33060827 PMC7566602

[41] Sun R Wang Z Zhao J Ren P Ma J Guo Y. Optimized detection of unknown MET exon 14 skipping mutations in routine testing for patients with non-small-cell lung cancer. JCO Precis Oncol. (2023) 7:e2200482. doi: 10.1200/PO.22.00482, PMID: 36848606

[42] Buckley PT Lee MT Sul JY Miyashiro KY Bell TJ Fisher SA. Cytoplasmic intron sequence-retaining transcripts can be dendritically targeted via ID element retrotransposons. Neuron. (2011) 69:877–84. doi: 10.1016/j.neuron.2011.02.028, PMID: 21382548 PMC3065018

[43] Belgrader P Cheng J Zhou X Stephenson LS Maquat LE. Mammalian nonsense codons can be cis effectors of nuclear mRNA half-life. Mol Cell Biol. (1994) 14:8219–28. doi: 10.1128/mcb.14.12.8219-8228.1994 7969159 PMC359361

[44] Lee SY Jin CC Choi JE Hong MJ Jung DK Do SK. Genetic polymorphisms in glycolytic pathway are associated with the prognosis of patients with early stage non-small cell lung cancer. Sci Rep. (2016) 6:35603. doi: 10.1038/srep35603, PMID: 27767175 PMC5073284

[45] Chen Y Zhang H Xu A Li N Liu J Liu C. Elevation of serum l-lactate dehydrogenase B correlated with the clinical stage of lung cancer. Lung Cancer. (2006) 54:95–102. doi: 10.1016/j.lungcan.2006.06.014, PMID: 16890323

[46] Koh YW Lee SJ Park SY. Prognostic significance of lactate dehydrogenase B according to histologic type of non-small-cell lung cancer and its association with serum lactate dehydrogenase. Pathol Res Pract. (2017) 213:1134–8. doi: 10.1016/j.prp.2017.07.006 28756978

[47] Deng H Gao Y Trappetti V Hertig D Karatkevich D Losmanova T. Targeting lactate dehydrogenase B-dependent mitochondrial metabolism affects tumor initiating cells and inhibits tumorigenesis of non-small cell lung cancer by inducing mtDNA damage. Cell Mol Life Sci. (2022) 79:445. doi: 10.1007/s00018-022-04453-5, PMID: 35877003 PMC9314287

[48] Millares L Barreiro E Cortes R Martinez-Romero A Balcells C Cascante M. Tumor-associated metabolic and inflammatory responses in early stage non-small cell lung cancer: local patterns and prognostic significance. Lung Cancer. (2018) 122:124–30. doi: 10.1016/j.lungcan.2018.06.015 30032820

[49] Cao L Hong W Cai P Xu C Bai X Zhao Z. Cryptotanshinone strengthens the effect of gefitinib against non-small cell lung cancer through inhibiting transketolase. Eur J Pharmacol. (2021) 890:173647. doi: 10.1016/j.ejphar.2020.173647, PMID: 33049304

[50] Shan C Kang HB Elf S Xie J Gu TL Aguiar M. Tyr-94 phosphorylation inhibits pyruvate dehydrogenase phosphatase 1 and promotes tumor growth. J Biol Chem. (2014) 289:21413–22. doi: 10.1074/jbc.M114.581124, PMID: 24962578 PMC4118105

[51] Warburg O Wind F Negelein E. The metabolism of tumors in the body. J Gen Physiol. (1927) 8:519–30. doi: 10.1085/jgp.8.6.519, PMID: 19872213 PMC2140820

[52] Akram M. Mini-review on glycolysis and cancer. J Cancer Educ. (2013) 28:454–7. doi: 10.1007/s13187-013-0486-9 23728993

[53] Li XB Gu JD Zhou QH. Review of aerobic glycolysis and its key enzymes-new targets for lung cancer therapy. Thorac Cancer. (2015) 6:17–24. doi: 10.1111/1759-7714.12148, PMID: 26273330 PMC4448463

[54] Reiter RJ Sharma R Ma Q. Switching diseased cells from cytosolic aerobic glycolysis to mitochondrial oxidative phosphorylation: a metabolic rhythm regulated by melatonin? J Pineal Res. (2021) 70:e12677. doi: 10.1111/jpi.12677, PMID: 32621295

[55] Schmidt C Sciacovelli M Frezza C. Fumarate hydratase in cancer: a multifaceted tumour suppressor. Semin Cell Dev Biol. (2020) 98:15–25. doi: 10.1016/j.semcdb.2019.05.002, PMID: 31085323 PMC6974395

